# Association analysis and *in silico* functional predictions of RMDN2 variants in chickens

**DOI:** 10.5713/ab.250758

**Published:** 2026-03-11

**Authors:** Ashi Li, Yuechen Liao, Yangqiwen Luo, Runbang Zhu, Cangning Zhang, Xingguo Wang, Meng Ma, Liumei Sun, Liang Qu, Manman Shen

**Affiliations:** 1 Jiangsu Key Laboratory of Sericultural and Animal Biotechnology, School of Biotechnology, Jiangsu University of Science and Technology, Zhenjiang, China; 2 Key Laboratory of Silkworm and Mulberry Genetic Improvement, Ministry of Agriculture and Rural Affairs, Sericultural Scientific Research Center, Chinese Academy of Agricultural Sciences, Zhenjiang, China; 3 Jiangsu Institute of Poultry Science, Yangzhou, China

**Keywords:** Chicken, Egg Production, Phylogenetic Tree Analysis, *RMDN2*, Single-nucleotide Polymorphism

## Abstract

**Objective:**

Microtubule dynamics regulator protein 2 (*RMDN2*) plays a crucial role in cell division, cytoskeleton maintenance, and various cellular processes, thereby establishing it as a candidate gene influencing chicken follicle development in our previous studies. This research aims to explore single-nucleotide polymorphisms (SNPs), perform phylogenetic analysis, and assess sequence characteristics of RMDN2, offering valuable insights for molecular marker-assisted breeding and enhancing the understanding of its regulatory mechanisms.

**Methods:**

SNPs within the *RMDN2* coding sequence region were identified in an F_2_ resource population. Bioinformatics tools were employed to investigate the effects of SNP mutations on the structure and function of RMDN2 protein. Additionally, a phylogenetic tree was constructed to elucidate the potential mechanisms underlying the role of RMDN2 in chicken laying traits.

**Results:**

Four novel exonic SNPs were identified: SNP1 (c.250G>A, p.Val84Ile), SNP2 (c.270G>C, p.Lys90Asn), SNP3 (c.533G>T, p.Gly178Val), and SNP4 (c.606G>A). The heterozygous genotypes of SNP1, SNP3, and SNP4 showed a significant association with increased egg number at 66 weeks (p<0.05). In contrast, the heterozygous genotype of SNP2 is associated with higher body weight at first egg (BWFE) (p<0.05). Notably, the H1H1 haplotype combination demonstrated a significant association with reduced BWFE, body weight at 105 days, and first egg weight (p<0.05). Missense mutations in SNP1, SNP2, and SNP3 may influence the hydrophilic/hydrophobic properties, transmembrane regions, functional domains, and secondary structure of the RMDN2 protein, potentially reducing its stability. Phylogenetic analysis demonstrated complete sequence homology between chicken and quail, indicating substantial conservation within species of the same order, while showing a marked decrease across different taxonomic orders.

**Conclusion:**

These findings enrich the candidate gene pool associated with the regulation of laying traits in chickens. However, further validation through *in vivo* and *in vitro* experiments remains necessary to strengthen the theoretical foundation for molecular breeding strategies.

## INTRODUCTION

Regulator of Microtubule Dynamics 2 (*RMDN2*) encodes a key regulator of microtubule dynamics, which is essential for cell division and cytoskeletal homeostasis. It is broadly expressed across various tissues and belongs to the microtubule-associated protein family. Current research has linked RMDN2 to human diseases, particularly cancer, suggesting that it may function as a cancer-promoting driver with potential diagnostic, prognostic, and therapeutic value [1]. Moreover, a genome-wide association study identified a significant association between the single nucleotide polymorphism (SNP) rs2113389 at the RMDN2-CYP1B1 locus and Alzheimer’s disease [2]. Despite these findings, the genetic variation and biological function of *RMDN2* remain poorly characterized, especially in animals.

*RMDN2* shares homology with Regulator of Microtubule Dynamics 3 *(RMDN3*, *PTPIP51)*, a mitochondrial protein that mediates lipid radical transfer from mitochondria to the endoplasmic reticulum to mitigate oxidative stress [3]. While *RMDN3* has been relatively well studied, the structural characteristics, specific roles, and gene structure of *RMDN2* in chickens remain unexplored. Our previous study identified *RMDN2* as a candidate gene influencing chicken follicle development, with expression levels increasing during follicular development and peaking in F1 follicles [4], suggesting a potential role in ovarian function and egg-laying performance.

Molecular marker-assisted selection is an effective strategy for enhancing the efficiency of egg production breeding. SNPs are ideal molecular markers due to their high density, low mutation rates, stable inheritance biallelic polymorphism, and strong representativeness [5]. Numerous SNPs associated with laying traits have been identified in poultry, including variants in the RFamide-related peptide (*RFRP*) gene in Zhenning Yellow chickens [6], prolactin (*PRL*) gene in chickens [7], and tyrosine aminotransferase (*TAT*) gene in Muscovy ducks [8]. These findings support the potential of SNPs as effective molecular markers for improving egg production. Given this, the continued discovery of novel SNPs promises to provide valuable tools for breeding. In our preliminary study, we identified four mutations in the coding sequence region of the *RMDN2* gene through pooled sequencing. However, the relationship between these *RMDN2* gene variants and laying performance remains unclear and warrants further investigation.

Mutations in key genes can markedly influence important phenotypic traits, and bioinformatics approaches offer powerful tools to investigate these relationships. For instance, polymorphism analyses of Cytochrome P450 Family 11 Subfamily A Member 1 (CYP11A1) in sheep have linked specific variants to litter size [9]. Similarly, a missense mutation in growth differentiation factor 9 (GDF9) is predicted to alter the protein’s tertiary structure and affect follicle-stimulating factor activity, highlighting its potential as a genetic marker for improving sheep litter size [10]. In Nigerian sheep, computational assessment of Prion Protein (PRNP) polymorphisms using PolyPhen-2 and PROVEAN suggested a deleterious effect for p.Arg154His, in contrast to the benign p.His171Gln [11]. Further, structural and functional predictions for the Melatonin Receptor 1A (*MTNR1A*) gene in Indonesian fine-tailed ewes indicated that the p.Val127Ile SNP may severely disrupt protein structure and stability, correlating with litter size variation [12]. Collectively, these studies demonstrate how bioinformatics tools can effectively prioritize putative causal SNPs and interpret their functional implications for complex traits.

In this study, we examined the association between polymorphisms in the RMDN2 gene and reproductive performance in an F_2_ population generated from a cross between Rhode Island White and White Leghorn chickens. Furthermore, bioinformatics analyses were performed to preliminarily investigate the potential functional and regulatory roles of RMDN2. The results of this work will contribute to a theoretical framework that can guide molecular-assisted breeding strategies for enhancing egg production performance.

## MATERIALS AND METHODS

### Genomic DNA samples collection for polymorphism analysis

The experimental chickens were obtained from an F_2_ resource population, which was constructed by the Jiangsu Institute of Poultry Science through intercrossing Rhode Island White and White Leghorn breeds with the objective of developing a high-yield chicken strain. The following performance traits were recorded: body weight at 42 days (BW42), body weight at 105 days (BW105), body weight at first egg (BWFE), body weight at 280 days (BW280), age at first egg (AFE), egg number at 40 weeks (EN40), egg number at 66 weeks (EN66), weight at the first egg (WFE), egg weight at 280 days (EW280). Blood samples were collected from the wing vein and stored in EDTA-containing anticoagulant tubes at 4°C until DNA extraction. Finally, a total of 149 hens were used for genotypes.

### DNA extraction, polymerase chain reaction and DNA sequencing

Genomic DNA was extracted from blood using phenol-chloroform extraction, dissolved in TE buffer (10 mM Tris-HCl, 1 mM EDTA, pH 8.0), and stored at −20°C after quality assessment with an Agilent Bioanalyzer 2100 system (Agilent Technologies). Nine primer pairs (P1–P9) targeting the exonic regions of *RMDN2* (GenBank accession: NC_052534.1) were designed using Primer Premier 5.0, with sequences provided in Supplement 1. Annealing temperatures were optimized through gradient polymerase chain reaction (PCR). PCR amplification was performed using 2× Master Mix (Vazyme Biotech) in 40 μL reactions containing 20 μL High-Fidelity DNA Polymerase (AG Scientific), 2 μL each of forward and reverse primers (10 μM), 2 μL genomic DNA (10 ng/μL), and 14 μL nuclease-free water. The reaction protocols followed the 2× Master Mix instructions. PCR products were validated by 1.5% agarose gel electrophoresis. All validated PCR products were sequenced using Sanger sequencing at Sangon Biotech. Sequence variants were identified and analyzed using SnapGene 6.0 (Insightful Science). Genotyping was performed using Chromas software.

### Single-nucleotide polymorphisms and association analysis

Allele, genotype frequencies, Hardy-Weinberg equilibrium (HWE), gene heterozygosity (He), effective allele numbers (Ne), and polymorphism information content (PIC) were analyzed using POPGENE v 1.3. Haplotype frequencies were estimated with PHASE v 2.1, employing Bayesian algorithms with 1,000 iterations and 500 burn-in cycles. Linkage disequilibrium (LD) was analyzed using Haploview v4.2, with LD strength quantified by both D′ and r^2^ metrics. Associations between genetic variants (SNPs/haplotypes) and laying performance were evaluated using generalized linear models (GLM) in SPSS v20.0, with the following model:


(1) $${\text{Y}}_{\text{ij}}=\mu +{\text{G}}_{\text{i}}+{\text{e}}_{\text{ij}}$$

where Y_ij_ is the phenotype of each hen, μ is the population mean, G_i_ is the effect of genotype or haplotype, and e_ij_ is the random error effect. The continuous variables were represented as mean±standard deviation (SD) and p<0.05 was significant.

### Phylogenetic tree construction of RMDN2

The protein sequences of RMDN2 were retrieved from the NCBI database for a diverse set of species. A phylogenetic tree was subsequently constructed using MEGA 11.0 software.

### Prediction of the effects of mutations on the structure of RMDN2

The physicochemical and structural characteristics of the chicken RMDN2 protein, encompassing attributes such as hydrophilicity/hydrophobicity, signal peptides, glycosylation and phosphorylation sites, transmembrane regions, domains, and secondary structure, were predicted utilizing the online bioinformatics tools presented in Supplement 2. Subsequently, the tertiary structure of the wild-type RMDN2 protein was modeled using SWISS-MODEL, capitalizing on recent advancements in computational prediction that have significantly enhanced the interpretation of SNP effects in animal breeding [13]. For the identified amino acid substitutions, PyMOL v1.0 was employed to visualize and analyze the structural ramifications of individual mutations. Given the limitations of any single algorithm in reliably predicting the functional consequences of missense SNPs, a combined methodological approach was adopted. The possible impacts on protein function were evaluated using SIFT, PANTHER, and PolyPhen-2, while potential alterations in structural stability at mutation sites were predicted with I-Mutant2.0, mCSM, and MUpro.

## RESULTS

### *RMDN2* gene polymorphisms

**Figure 1:**
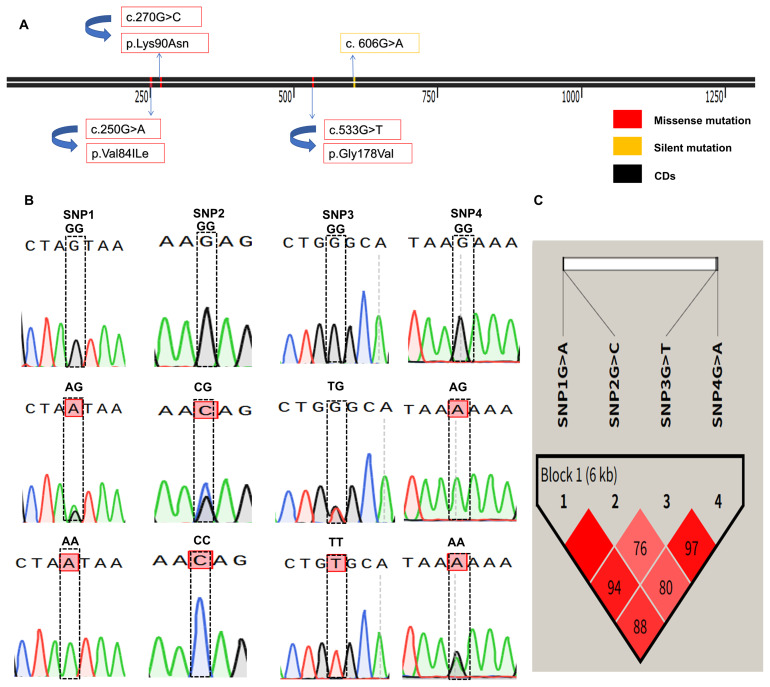
*RMDN2* gene polymorphism analysis. (A) Schematic diagram of CDS region mutations in *RMDN2* gene; (B) Polymorphic base mutation types of the *RMDN2* gene; (C) Linkage disequilibrium analysis of *RMDN2* gene polymorphisms in the F_2_ resource population. The value in the box indicates the linkage disequilibrium value (D′) of the single nucleotide polymorphism, which will not be displayed when D′ = 1. The darker the red color of the box, the stronger the linkage disequilibrium. *RMDN2*, Regulator of Microtubule Dynamics 2.

The polymorphisms of *RMDN2* were presented in Supplement 3 and Figure 1A. Four SNPs were identified in this population, with all except SNP4 resulting in amino acid substitutions. Specifically, SNP1 (chr3:31467574, G>A, p.Val84Ile) and SNP2 (chr3:31467594, G>C, p.Lys90Asn) are located in exon 1, while SNP3 (chr3:31474246, G>T, p.Gly178Val) and SNP4 (chr3:31474319, G>A) are located in exon 2. Each SNP locus has three genotypes (Figure 1B). LD analysis of these four SNPs exhibited D′ values ranging from 0.76 to 1.00, indicating strong LD among these mutations (Figure 1C). All SNPs were in HWE (p>0.0*5*, Table 1). Table 1 further shows that the observed Heterozygosity (Ho) and expected Heterozygosity (He) at each locus ranged from 0.25 to 0.51 and 0.25 to 0.50, respectively. The PIC ranged from 0.20 to 0.38, and the effective Ne ranged from 1.3 to 2.0. These findings suggest that *RMDN2* gene loci exhibit low to moderate polymorphism.

### Association analysis between polymorphisms and reproductive traits

The associated between *RMDN2* polymorphisms and reproductive traits are summarized in Table 2. For SNP1, the AG genotype was associated with higher EN40 and EN66 values compared to the GG genotype (p<0.05). For SNP2, chickens with the CG genotype exhibited higher BWFE values than those with the CC genotype (p<0.05). For SNP3, chickens with the TG genotype had higher EN66 values compared to the GG genotype (p<0.05). For SNP4, chickens with the AG and GG genotypes outperformed those with the AA genotype in EN40 and EN66 (p<0.05).

### Haplotype analysis of single nucleotide polymorphisms

Haplotype analysis was performed, excluding haplotypes with frequencies below 1% from further analysis, which resulted in five haplotypes, labeled H1 to H5 (Table 3). Association analysis between haplotype combinations and laying traits revealed that the BWFE and BW105 of the H1H1 combination were significantly lower than those of H1H3 (p<0.05) (Table 4). Additionally, the FEW of H2H2 and H1H4 was significantly higher than those of H1H1 (p<0.05). Furthermore, the FEW of H2H2 was significantly higher than those of H1H2, H1H3, and H2H3 (p<0.05). Notably, the H1H4 haplotype, characterized by three heterozygous genotypes at SNP1, SNP2, and SNP3, along with a homozygous genotype at SNP4, exhibited the highest EN66 among the groups. However, its allele frequency was only 2.16% in the current population.

### Bioinformatics analysis of wild-type and mutant RMDN2 protein

**Figure 2:**
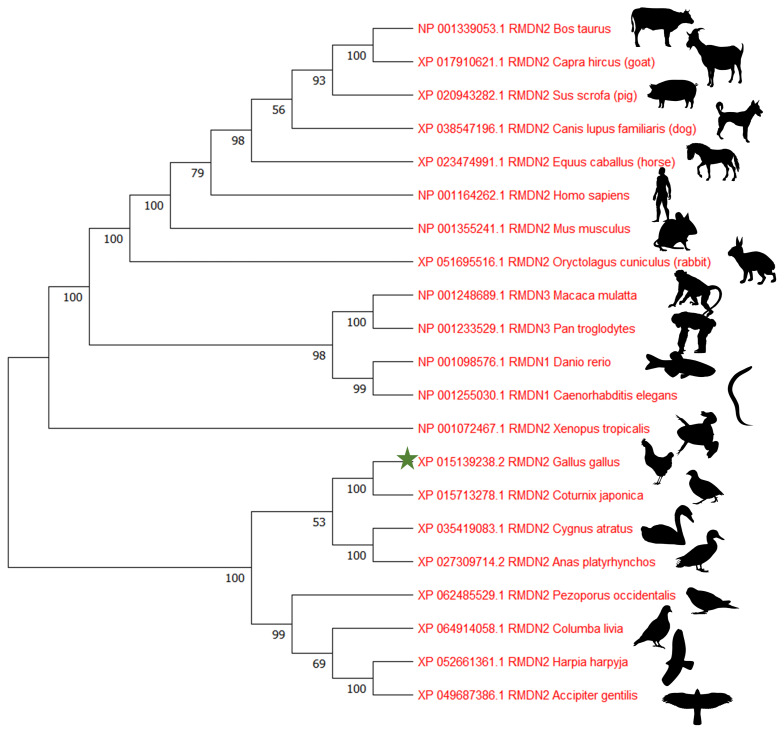
Systematic purification tree construction of RMDN2 protein. RMDN2, Regulator of Microtubule Dynamics 2. The asterisk indicates the primary research focus on chicken RMDN2.

Bioinformatics analysis of wild-type and mutant RMDN2 proteins was conducted to study the effects of SNP1, SNP2, and SNP3. As shown in Figure 2, there was 100% sequence homology between chicken and quail, duck and swan, sheep and cow, monkey and chimpanzee, and eagle and hawk. Caenorhabditis elegans and zebrafish showed 99% homology, sheep and cattle compared with pigs showed 93%, and primates compared with more distantly related species showed 98%. Galliformes and Anseriformes had 53% homology. Overall, these results suggest that RMDN2 homology is high within species of the same order but shows limited sequence conservation across species from different orders.

**Figure 3:**
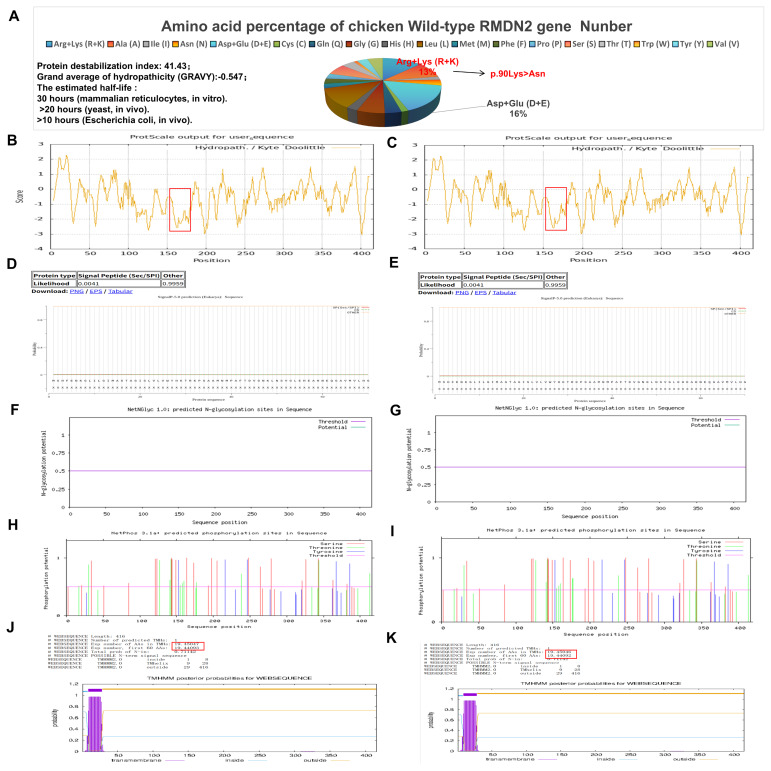
Bioinformatics analysis of wild-type and mutant RMDN2 protein. (A) Amino acid composition of RMDN2 protein; (B, C) Hydrophilicity-hydrophobicity analysis of wild-type and mutant RMDN2 protein (Positive values represent hydrophobicity and negative values represent hydrophilicity.); (D, E) Prediction of signal peptides of wild-type and mutant RMDN2 protein; (F, G) Prediction of glycosylation of wild-type and mutant RMDN2 protein; (H, I) Prediction of phosphorylation sites of wild-type and mutant RMDN2 protein; (J, K) Prediction of transmembrane regions of wild-type and mutant RMDN2 protein. RMDN2, Regulator of Microtubule Dynamics 2. The red box is used to emphasize that SNP variation results in region changes of secondary structure.

The predicted physicochemical properties and hydrophilicity/hydrophobicity analysis indicated that the instability index of the chicken RMDN2 protein is 41.43, with an average hydrophobicity value of −0.547, indicating that RMDN2 is likely an unstable and hydrophilic protein under *in silico* conditions (Figure 3A, Table 5). The RMDN2 protein comprises 416 amino acids. The SNP2 mutation was predicted to reduce the total positive charge of amino acid residues (Arg+Lys). These SNP also cause a slight decrease in the isoelectric point, while marginally enhancing the stability and hydrophobicity of the protein. Notably, the hydrophilic/hydrophobic positions within the 150–200 amino acid region shifts upwards, indicating an increase in overall hydrophobicity. (Figures 3B, 3C). Analysis using SignalP 5.0, NetNGlyc 1.0, and NetOGlyc 4.0 revealed that the RMDN2 protein lacks signal peptides and glycosylation sites (Figures 3D, 3F). Furthermore, a total of 39 potential phosphorylation sites were identified using NetPhos 3.1, including 12 serine, 22 threonine, and 5 tyrosine phosphorylation sites (Figure 3H). The mutation sites of these SNPs did not affect the signal peptide, glycosylation sites, or phosphorylation sites of RMDN2 (Figures 3E, 3G, 3I). Analysis of transmembrane regions indicated that RMDN2 contains a transmembrane domain spanning approximately 19 amino acids, located between positions 9 and 28 (Figures 3J, 3K), suggesting it may be a transmembrane protein.

**Figure 4:**
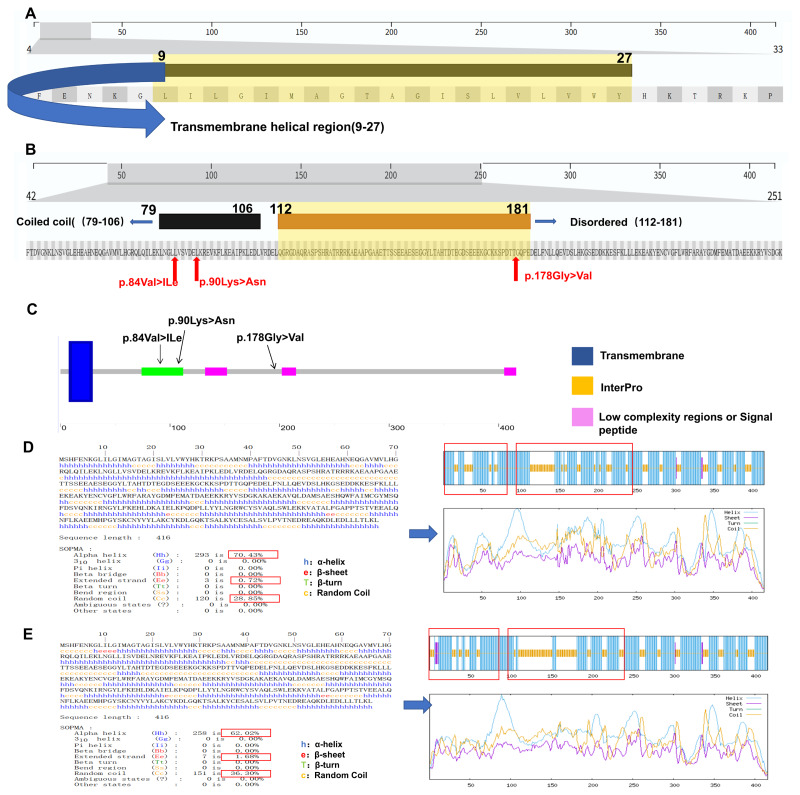
RMDN2 protein region and secondary structure prediction. (A, B) Prediction of structural regions of RMDN2 protein; (C) Prediction of functional regions of RMDN2 protein; (D, E) Prediction of secondary structure of wild-type and mutant RMDN2 protein. RMDN2, Regulator of Microtubule Dynamics 2. The red box is used to emphasize that SNP variation results in region changes of secondary structure.

Moreover, UniProt predictions indicate that the RMDN2 protein may contain a coiled-coil domain (residues 79–106) and a disordered region (residues 112–181) within the amino acid sequence spanning residues 42–251 (Figures 4A, 4B), both of which could potentially be affected by the three identified missense SNPs. Domain prediction using the SMART online software further identified that SNP1 (p.Val84Ile) and SNP2 (p.Lys90Asn) may influence functional domains, including enzyme active sites and binding sites (Figure 4C). A comparative analysis of the secondary structure of the RMDN2 protein, conducted with and without missense mutations using SOPMA, revealed alterations (Figures 4D, 4E). Specifically, the mutated RMDN2 protein exhibited an increased proportion of random coils and β-sheets, accompanied by a decrease in α-helix. Notably, β-sheet appeared within the transmembrane region. In the sequence region spanning residues 100–150, the unmutated protein predominantly exhibited α-helical structures, whereas the mutated protein showed an increased presence of random coil.

### Pros and cons of mutations in RMDN2

**Figure 5:**
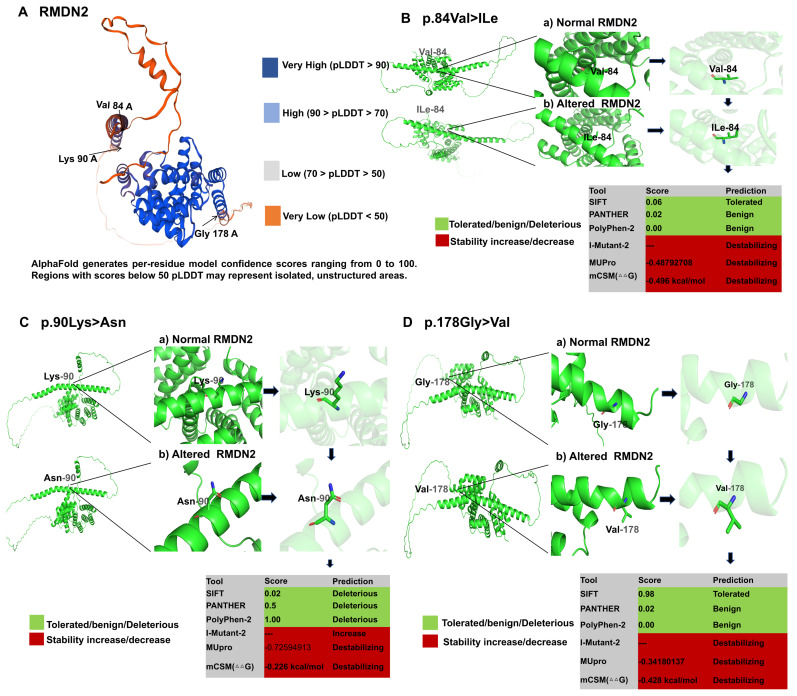
3 SNPs Pros and cons for RMDN2 functionality. (A) Prediction of RMDN2 protein tertiary structure (modeling); (B–D) Potential deleterious effects of missense mutations of p.84Val>ILe, p.90Lys>Asn and p.178Gly>Val on RMDN2. The 3D structure is shown as cartoons encrypted within a transparent surface. RMDN2, Regulator of Microtubule Dynamics 2; SNP, single nucleotide polymorphism.

To investigate the possible impact of single amino acid mutations on RMDN2 protein, six advanced computational tools (SIFT, PANTHER, PolyPhen-2, I-Mutant2, mCSM, and MUpro) were used to assess. The analysis focus was on the protein’s structure, function, stability, and biological activity, with results visualized via SWISS-MODEL and PyMol-v1. The modeling results (Figure 5A) suggest that RMDN2, modeled as a monomer, showed pLDDT values between 50–90 for the mutations p.84Val>ILe, p.90Lys>Asn, and p.178Gly>Val, indicating moderate confidence. Tools predicted that p.90Lys>Asn might be highly detrimental, with mCSM and MUpro suggesting a decrease in protein stability, while I-Mutant2 indicated a potential increase in stability (Figure 5C). Conversely, p.84Val>ILe (Figure 5B) and p.178Gly>Val (Figure 5D) were not predicted to be harmful but were linked to a reduction in stability.

## DISCUSSION

In poultry production, reproductive traits like egg production, egg weight, and AFE are crucial economic indicators. This study examined F_2_ resource populations derived from two high-yielding egg-laying chicken breeds subjected to long-term intensive selection for improved laying performance. Four novel SNPs within the RMDN2 gene were identified, with genetic diversity at these loci being moderate to low. Prolonged selective breeding typically leads to the reduction of genetic diversity [14], this mechanism is primarily achieved through selective sweeps, whereby the fixation of a beneficial mutation is accompanied by a reduction in genetic diversity in the surrounding genomic region [15,16]. Interestingly, the wide-type GG and GG genotypes of SNP1 and SNP3 showed lower frequencies and were associated with the lower EN66 compared to other genotypes. As a result of long-term selection, the population likely experienced genetic bottlenecks due to the elimination of individuals with lower egg production. It is hypothesized that RMDN2 may have been indirectly selected during breeding programs, with its genetic variations linked to the enhancement of egg production. However, the current findings suggest that the utility of this marker for future breeding efforts is limited in this population, as the EN66 values of the alternative genotypes are close to each other. Combined genotypic effects of multiple SNPs, particularly those involving heterozygous configurations, can significantly influence egg production traits through cooperative interactions [17]. The individual effects of SNPs are often small compared with their combined effects, highlighting the importance of haplotype-level analysis in molecular marker-assisted selection [18]. The common heterogeneity observed in this quantitative trait supports the potential of hybrid breeding to enhance egg production [19]. Future studies should increase the sample size and employ whole-genome analysis to incorporate multiple genetic loci, alongside environmental factors, to gain a deeper understanding of their roles in egg production traits. Such research will provide a more comprehensive reference for the genetic improvement of other chicken populations.

Given that the functional relationship between genetic markers and trait phenotypes cannot be effectively captured by a single SNP, LD and haplotype analysis were employed to investigate the association between these loci and reproductive traits [20]. The H1H1 haplotype combination, derived from the three SNPs on the *PRKCA* gene, was advantageous for enhancing eggshell strength and thickness in female ducks [21]. Similarly, the H1H1 combination on the *MC1R* gene was effective in increasing the black plumage proportion in Guangxi Yao chickens [22]. In the present study, the H1H1 combination was associated with low values of BWFE, BW105, and FEW, whereas the H2H2 and H1H4 combinations were associated with elevated FEW values. Selection for a lighter body weight in chicken breeding lines may enhance reproductive performance, as individuals with earlier AFE and lighter FEW tend to have higher total egg number [23]. Notably, the H1H4 combination, formed by heterozygous genotypes of SNP1, SNP2, SNP3 and the GG genotype of SNP4, showed the highest EN66, despite having the lowest allele frequency. This observation may be explained by heterosis, which is common in quantitative traits and supports the potential of hybrid breeding for improving egg production [24]. However, future studies should explore the beneficial effects of heterozygous haplotypes on reproductive traits by increasing sample sizes, in order to fully assess and leverage the breeding potential of the H1H4 combination.

The analysis of physicochemical properties analysis indicates that the missense mutation induces subtle but potentially meaningful changes in the biochemical characteristics of RMDN2, which may collectively influence its structure and function. Alteration in amino acid composition can modify charge distribution and hydrophobicity, both of which are critical determinants of protein folding and molecular interactions [25]. These effects may modulate interaction efficiency without causing complete loss of function. Hydrophobicity plays a central role in stabilizing protein structure and directing secondary structure formations [26]. Previous studies have demonstrated that increased hydrophobic character can enhance core packing and thermal stability [27], but it may also predispose proteins to adopt β-sheet–rich conformations under specific conditions [28]. These shifts can influence functional dynamics, especially for proteins requiring structural flexibility. Protein stability represents a balance between maintaining structural integrity and preserving conformational adaptability [29]. Excessive stabilization may restrict the dynamic motions required for catalysis or regulation [30]. Therefore, even minor stability changes may have disproportionate functional consequences. Overall, the mutation may act as a fine-tuning factor, subtly influencing folding behavior, interaction networks, and functional regulation.

Further *in silico* analysis revealed that RMDN2 lacks signal peptide and glycosylation sites, but it may function as a transmembrane protein, consistent with its role within the RMDN family [3]. Transmembrane regions are typically composed of α-helices. Although the hydrophobic distribution within this region (amino acids 9–27) remains largely unchanged, the introduction of β-sheets may influence helical continuity, potentially affecting the transmembrane insertion process [31]. Therefore, the presence of β-sheets in the RMDN2 transmembrane region might similarly expose hidden phosphorylation sites or ligand-binding motifs, potentially impacting signal transduction. A decrease in α-helix content can affect the hydrophobic interface, inhibiting protein oligomerization and affecting functions like signal transduction or cell adhesion [32]. Disordered regions often have post-translational modification sites and are involved in dynamic protein interactions [33]. The primary function of the coiled coil is to mediate protein oligomerization and ligand binding [34]. SNP1 and SNP2 mutations located in the coiled-coil region (79–106 residues), while SNP3 mutation located the disordered region (residues 112–181), lead to a reduction in α-helix and an increase random coil and β-sheet, implying that these mutations may influence the post-translational function of RMDN2 and its role in signal transduction. RMDN2, as a member of RMDN family, is involved in microtubule dynamics, which are critical for chromosome pairing and cell division [35]. Hence, mutations in RMDN2 could potentially influence the post-translational function and signal transduction involved in microtubule dynamic process. However, the precise advantages and disadvantages of RMDN2 need to be explored through experiment validation in future studies.

Additionally, we observed that the mutant allele frequency was higher than that of the wild type, and the EN66 of the homozygous mutant was close to that of the heterozygous genotype. This suggests that the SNP mutations in RMDN2 are unlikely to impair its function. On the contrary, mutations may enable RMDN2 to more actively affect ovarian function, thus contributing to increased egg production.

Phylogenetic analysis of RMDN2 demonstrated complete sequence homology between chicken and quail, with notable conservation observed among species within the same order. However, homology significantly decreased across different taxonomic orders, indicating that species from distinct orders may exhibit similar evolutionary responses to environmental changes [36]. This phenomenon is also observed universally when employing marker genes in studies of species evolution [37,38]. Our findings suggest that RMDN2 could serve as a valuable tool for comparative evolutionary studies across species.

## CONCLUSION

This study investigated genetic polymorphisms within the CDS region of the *RMDN2* gene in an F_2_ chicken resource population, identifying four novel SNPs. The heterozygous genotypes of SNP1, SNP3, and SNP4 were associated with increased EN66, while the heterozygous genotype of SNP2 was associated with elevated BWFE. The H1H1 haplotype combination was associated with lower BWFE, BW105, and FEW. Missense mutations were predicted to influence the function of RMDN2 protein. Phylogenetic analysis demonstrated high conservation within species of the same order and limited sequence conservation across species of different orders. These findings contribute to the understanding of genetic factors influencing laying traits and provide a foundation for further exploration of the RMDN2 gene’s role in poultry. However, the limitations of the current study, including the small sample size and the lack of experimental validation, must be acknowledged, as they may affect the robustness and generalizability of the conclusions. Future studies, including larger sample sizes and experimental validation of the predicted functional effects, are necessary to confirm the role of RMDN2 in poultry breeding.

## Figures and Tables

**Table 1:** Genotype, allele frequencies and genetic diversity for the SNPs in *RMDN2*

SNP	Genotype frequency (n)			Allele gene frequency		X^2^ (HWE)	p-value^1)^	Genetic polymorphism			
	
	AA	AB	BB	A	B			PIC	He	Ne	Ho
c. 250G>A (SNP^1)^	3 (0.02)	39 (0.26)	107 (0.72)	0.151	0.849	0.041	0.840	0.222	0.255	1.342	0.255
c. 270G>C (SNP2)	33 (0.22)	76 (0.5^1)^	40 (0.27)	0.477	0.523	0.077	0.781	0.374	0.499	1.995	0.510
c. 533G>T (SNP3)	4 (0.03)	40 (0.27)	105 (0.70)	0.161	0.839	0.040	0.842	0.238	0.276	1.380	0.268
c. 606G>A (SNP4)	2 (0.0^1)^	39 (0.26)	108 (0.72)	0.140	0.850	0.644	0.422	0.230	0.258	1.348	0.262

1) Hardy–Weinberg equilibrium (HWE) test, with p>0.05 indicating that the population conforms to the HWE.

SNP, single-nucleotide polymorphism; *RMDN2*, Regulator of Microtubule Dynamics 2; PIC, polymorphism information content; He, expected heterozygosity; Ne, effective number of alleles; Ho, observed heterozygosity.

**Table 2:** Association between the polymorphism of *RMDN2* and the laying traits in the F_2_ resource population

Mutation locus	c. 250G>A (SNP1)			c. 270G>C (SNP2)			c. 533G>T (SNP3)			c. 606G>A (SNP4)		
Genotype	GG	AG	AA	GG	CG	CC	GG	TG	TT	GG	AG	AA
Allele frequency (%)	2.01	26.17	71.81	22.15	51.01	26.85	2.68	26.85	70.47	72.48	26.17	1.34
BW42 (g)	444.33± 50.36	450.66± 51.62	433.86± 58.64	441.44± 61.29	440.20± 57.19	432.58± 53.39	423.00± 17.53	451.97± 54.13	433.92± 58.30	434.99± 58.33	448.29± 53.70	435.00± 18.38
BW105 (g)	1,022.67± 98.65	1,108.91± 86.51	1,077.36± 97.09	1,082.63± 67.23	1,099.04± 101.12	1,063.71± 93.66	1,040.25± 97.75	1,110.23± 88.85	1,076.41± 96.43	1,077.69± 95.73	1,104.41± 95.30	1,055.00± 25.46
BWFE (g)	1,539.33± 126.03	1,489.45± 110.90	1,473.86± 127.08	1,489.03± 111.23^ab^	1,497.96± 126.22^a^	1,443.80± 131.14^b^	1,502.00± 137.24	1,494.85± 111.28	1,472.50± 126.84	1,470.92± 125.08	1,498.08± 114.53	1,567.00± 141.42
BW280 (g)	1,550.67± 38.03	1,688.28± 183.52	1,650.36± 148.31	1,641.36± 131.96	1,661.57± 152.41	1,654.23± 172.25	1,682.50± 241.66	1,690.23± 178.72	1,645.18± 145.74	1,644.64± 145.99	1,699.49± 186.03	1,591.00± 14.14
AFE (d)	142.00± 10.82	138.05± 7.42	138.79± 8.05	138.91± 8.04	138.59± 7.81	138.60± 8.16	139.75± 10.50	138.23± 7.30	138.79± 8.10	138.39± 7.76	138.64± 7.64	143.50± 10.61
FEW (g)	40.33± 4.51	36.39± 2.77	36.86± 4.84	37.35± 4.18	36.87± 3.98	35.82± 3.41	39.33± 4.90	36.60± 3.12	37.04± 5.06	36.92± 4.86	36.79± 3.13	41.00± 7.07
EW280 (g)	60.37± 7.34	58.80± 2.73	58.07± 4.47	58.66± 4.55	58.53± 3.97	57.67± 4.60	59.35± 6.36	59.03± 2.94	58.00± 4.43	58.09± 4.40	58.94± 3.15	57.63± 8.10
EN40 (No.)	112.67± 24.58^b^	127.08± 10.43^a^	125.42± 11.53^ab^	122.09± 15.08	125.85± 10.86	127.05± 11.22	117.25± 22.10	127.37± 10.45	125.24± 11.56	125.58± 11.57^a^	126.57± 10.27^a^	107.50± 31.82^b^
EN66 (No.)	254.67± 39.83^b^	278.32± 20.95^a^	278.96± 18.12^a^	279.13± 19.16	278.35± 18.91	279.31± 18.41	258.75± 33.21^b^	279.92± 21.25^a^	278.44± 18.03^a^	279.08± 18.03^a^	277.65± 21.09^a^	249.00± 53.74^b^

In this study, statistical significance was determined using generalized linear models (GLM), with values presented as mean±SD.

a,b Letters are employed to indicate significant differences among groups, with a denoting the highest mean. Identical letters suggest non-significance (p>0.05), while different letters indicate significance (p<0.05).

*RMDN2*, Regulator of Microtubule Dynamics 2; SNP, single nucleotide polymorphism; BW42, body weight at 42 days (BW42); BW105, body weight at 105 days; BWFE, body weight at first egg; BW280, body weight at 280 days; AFE, age at first egg; FEW, first egg weight; EW280, egg weight at 280 days; EN40, egg number at 40 weeks; EN66, egg number at 66 weeks; SD, standard deviation.

**Table 3:** *RMDN2* gene haplotype analysis

Haplotype No.	Haplotype	Frequency
H1	ACTG	0.503358
H2	AGTG	0.325236
H3	GGGA	0.129907
H4	GGGG	0.01419
H5	ACGA	0.010629

*RMDN2*, Regulator of Microtubule Dynamics 2.

**Table 4:** Association analysis of haplotype combination of *RMDN2* and production performance in the F_2_ resource population

Diplotypes combination		H1H1 (ACTG/ACTG)	H1H2 (ACTG/AGTG)	H1H3 (ACTG/GGGA)	H1H4 (ACTG/GGGG)	H2H2 (AGTG/AGTG)	H2H3 (AGTG/GGGA)
Allele frequency (%)		26.62	36.69	12.95	2.16	10.79	10.79
Body weight-related	BW42 (g)	435.54± 53.85	430.53± 57.16	456.65± 56.43	474.67± 47.17	442.64± 75.74	439.73± 51.19
	BW105 (g)	1,057.61± 103.87^b^	1,083.96± 99.71^ab^	1,126.25± 109.25^a^	1,119.00± 67.01^ab^	1,094.20± 65.91^ab^	1,083.17± 58.76^ab^
	BWFE (g)	1,437.51± 132.31^b^	1,490.35± 125.20^ab^	1,524.33± 140.70^a^	1,478.67± 74.65^ab^	1,486.93± 112.62^ab^	1,463.29± 89.29^ab^
	BW280 (g)	1,648.70± 176.83	1,646.20± 137.38	1,708.94± 197.61	1,604.67± 177.43	1,634.33± 98.90	1,666.53± 165.29
Egg production performance metrics	AFE (d)	137.92± 7.64	139.08± 8.06	138.11± 7.81	135.00± 4.58	137.93± 8.28	139.27± 7.69
	FEW (g)	35.68± 3.25^c^	36.55± 4.87^bc^	36.73± 2.85^bc^	41.11± 9.58^ab^	39.84± 6.75^a^	36.16± 3.18^bc^
	EW280 (g)	57.64± 4.75	58.07± 4.42	58.87± 1.94	61.03± 1.55	58.55± 4.07	57.64± 3.61
	EN40 (No.)	126.49± 11.46	124.27± 11.86	127.53± 8.39	134.67± 3.79	124.67± 11.70	121.40± 16.42
	EN66 (No.)	278.48± 18.73	278.51± 17.93	273.88± 23.28	292.33± 6.11	278.29± 19.06	280.36± 21.22

In this study, statistical significance was determined using generalized linear models (GLM), with values presented as mean±SD.

a–c Letters are employed to indicate significant differences among groups, with a denoting the highest mean. Identical letters suggest non-significance (p>0.05), while different letters indicate significance (p<0.05).

*RMDN2*, Regulator of Microtubule Dynamics 2; BW42, body weight at 42 days; BW105, body weight at 105 days; BWFE, body weight at first egg; BW280, body weight at 280 days; AFE, age at first egg; FEW, first egg weight; EW280, egg weight at 280 days; EN40, egg number at 40 weeks; EN66, egg number at 66 weeks; SD, standard deviation.

**Table 5:** Differential physicochemical properties of wild-type and mutant RMDN2 proteins

Physicochemical property	Wild-type values	Mutant values	Explanation of variance
Theoretical pI	5.47	5.41	Mutations lead to changes in charge distribution and a slight decrease in isoelectric point
Molecular weight	46,677.88 Da	46,719.92 Da	Amino acid substitutions result in a slight increase in molecular weight
Total number of positively charged residues (Arg+Lys)	54	53	Mutation results in a decrease of 1 positively charged residue
Formula	C2060H3247N559O641S18	C2062H3249N559O642S18	The number of C, H, and O atoms increases slightly, while the number of N and S atoms remains unchanged.
Total number of atoms	6,525	6,530	
Instability index	41.43	41.06	Mutation results in a slightly more stable protein
Aliphatic index	77.69	78.63	Mutation causes a slight increase in the hydrophobicity of the protein
Grand average of hydropathicity (GRAVY)	−0.547	−0.534	

RMDN2, Regulator of Microtubule Dynamics 2.

## Data Availability

Upon reasonable request, the datasets of this study can be available from the corresponding author.

## SUPPLEMENTARY MATERIAL

**Supplement 1.** Sequence information of primers used in this study.

**Supplement 2.** Bioinformatics software-related information.

**Supplement 3.** Polymorphisms identified in the *RMDN2* gene.

## References

[1] Jiang L Chen S Stinnett V Concomitance of a novel RMDN2-ALK fusion and an EML4-ALK fusion in a lung adenocarcinoma Cancer Genet 2021 258–9 18 22 10.1016/j.cancergen.2021.06.004 34233240

[2] Nho K Risacher SL Apostolova LG CYP1B1-RMDN2 Alzheimer’s disease endophenotype locus identified for cerebral tau PET Nat Commun 2024 15 8251 10.1038/s41467-024-52298-2 39304655 PMC11415491

[3] Shiiba I Ito N Oshio H ER-mitochondria contacts mediate lipid radical transfer via RMDN3/PTPIP51 phosphorylation to reduce mitochondrial oxidative stress Nat Commun 2025 16 1508 10.1038/s41467-025-56666-4 39929810 PMC11811300

[4] Li A Liao Y Li D Transcriptome profiling of granulosa cells during follicular development identifies RMDN2 polymorphisms associated with reproductive traits in chickens Theriogenology 2026 252 117775 10.1016/j.theriogenology.2025.117775 41330057

[5] Schipper M de Leeuw CA Maciel BAPC Prioritizing effector genes at trait-associated loci using multimodal evidence Nat Genet 2025 57 323 33 10.1038/s41588-025-02084-7 39930082

[6] Tan YG Xu XL Cao HY Mao HG Yin ZZ RFamide-related peptides’ gene expression, polymorphism, and their association with reproductive traits in chickens Poult Sci 2021 100 488 95 10.1016/j.psj.2020.11.024 33518101 PMC7858160

[7] Rohmah L Darwati S Ulupi N Khaerunnisa I Sumantri C Polymorphism of prolactin (PRL) gene exon 5 and its association with egg production in IPB-D1 chickens Arch Anim Breed 2022 65 449 55 10.5194/aab-65-449-2022 36643022 PMC9832302

[8] Ju X Wang Z Cai D TAT gene polymorphism and its relationship with production traits in Muscovy ducks (Cairina Moschata) Poult Sci 2023 102 102551 10.1016/j.psj.2023.102551 36972669 PMC10050636

[9] Hu WP Liu MQ Tian ZL Polymorphism, expression and structure analysis of key genes in the ovarian steroidogenesis pathway in sheep (Ovis aries) Vet Med Sci 2021 7 1303 15 10.1002/vms3.485 33780162 PMC8294399

[10] Wang F Chu M Pan L Polymorphism detection of GDF9 gene and its association with litter size in Luzhong mutton sheep (Ovis aries) Animals 2021 11 571 10.3390/ani11020571 33671790 PMC7926531

[11] Adeola AC Bello SF Abdussamad AM Polymorphism of prion protein gene (PRNP) in Nigerian sheep Prion 2023 17 44 54 10.1080/19336896.2023.2186767 36892181 PMC10012947

[12] Abuzahra M Al-Shuhaib MBS Wijayanti D Effendi MH Mustofa I Moses IB A novel p.127Val>Ile single nucleotide polymorphism in the MTNR1A gene and its relation to litter size in Thin-tailed Indonesian ewes Anim Biosci 2025 38 209 22 10.5713/ab.24.0187 38938032 PMC11725752

[13] Alwan IH Aljubouri TRS Al-Shuhaib MBS A novel missense SNP in the fatty acid-binding protein 4 (FABP4) gene is associated with growth traits in Karakul and Awassi sheep Biochem Genet 2024 62 1462 84 10.1007/s10528-023-10504-8 37640973

[14] Elferink MG Megens HJ Vereijken A Hu X Crooijmans RPMA Groenen MAM Signatures of selection in the genomes of commercial and non-commercial chicken breeds PLOS ONE 2012 7 e32720 10.1371/journal.pone.0032720 22384281 PMC3287981

[15] Restoux G Rognon X Vieaud A Managing genetic diversity in breeding programs of small populations: the case of French local chicken breeds Genet Sel Evol 2022 54 56 10.1186/s12711-022-00746-2 35922745 PMC9347113

[16] Saravanan KA Panigrahi M Kumar H Bhushan B Dutt T Mishra BP Selection signatures in livestock genome: a review of concepts, approaches and applications Livest Sci 2020 241 104257 10.1016/j.livsci.2020.104257

[17] Kawata N Tsuchiya N Horikawa Y Two survivin polymorphisms are cooperatively associated with bladder cancer susceptibility Int J Cancer 2011 129 1872 80 10.1002/ijc.25850 21154810

[18] Ali MY Faruque S Ahmadi S Ohkubo T Genetic analysis of HSP70 and HSF3 polymorphisms and their associations with the egg production traits of Bangladeshi hilly chickens Animals 2024 14 3552 10.3390/ani14243552 39765456 PMC11672713

[19] Jiang Y Li X Liu J Genome-wide detection of genetic structure and runs of homozygosity analysis in Anhui indigenous and Western commercial pig breeds using PorcineSNP80k data BMC Genomics 2022 23 373 10.1186/s12864-022-08583-9 35581549 PMC9115978

[20] Georges M Charlier C Hayes B Harnessing genomic information for livestock improvement Nat Rev Genet 2019 20 135 56 10.1038/s41576-018-0082-2 30514919

[21] Tan GH Li JZ Zhang YY You MF Liao CM Zhang YG Association of PRKCA expression and polymorphisms with layer duck eggshell quality Br Poult Sci 2021 62 8 16 10.1080/00071668.2020.1817329 32893664

[22] Li J Wu R Wang Y A selection breeding pattern for sexually dimorphic breast plumage color in Guangxi Yao chickens Poult Sci 2024 103 104218 10.1016/j.psj.2024.104218 39190997 PMC11396058

[23] Chen A Zhao X Wen J Genetic parameter estimation and molecular foundation of chicken egg-laying trait Poult Sci 2024 103 103627 10.1016/j.psj.2024.103627 38593551 PMC11015155

[24] Cheng X Li X Yang M Genome-wide association study exploring the genetic architecture of eggshell speckles in laying hens BMC Genomics 2023 24 704 10.1186/s12864-023-09632-7 37993775 PMC10666442

[25] Tokuriki N Tawfik DS Stability effects of mutations and protein evolvability Curr Opin Struct Biol 2009 19 596 604 10.1016/j.sbi.2009.08.003 19765975

[26] Ravichandran A Puri A Bhate SH Habibullah BI Singh G Das R Structure-guided engineering of protein stability through core hydrophobicity Protein Sci 2025 34 e70360 10.1002/pro.70360 41230880 PMC12613165

[27] Yoda T Sugita Y Okamoto Y Hydrophobic core formation and dehydration in protein folding studied by generalized-ensemble simulations Biophys J 2010 99 1637 44 10.1016/j.bpj.2010.06.045 20816077 PMC2931739

[28] Sethuraman A Vedantham G Imoto T Przybycien T Belfort G Protein unfolding at interfaces: slow dynamics of α-helix to β-sheet transition Proteins 2004 56 669 78 10.1002/prot.20183 15281120

[29] DePristo MA Weinreich DM Hartl DL Missense meanderings in sequence space: a biophysical view of protein evolution Nat Rev Genet 2005 6 678 87 10.1038/nrg1672 16074985

[30] Havlásek M Marques SM Szotkowská V Decoding protein stabilization: impact on aggregation, solubility, and unfolding mechanisms J Chem Inf Model 2025 65 8688 701 10.1021/acs.jcim.5c00611 40768221 PMC12381856

[31] Zhmurov A Kononova O Litvinov RI Dima RI Barsegov V Weisel JW Mechanical transition from α-helical coiled coils to β-sheets in fibrin(ogen) J Am Chem Soc 2012 134 20396 402 10.1021/ja3076428 22953986 PMC3526676

[32] Mittal S Cai Y Nalam MNL Bolon DNA Schiffer CA Hydrophobic core flexibility modulates enzyme activity in HIV-1 protease J Am Chem Soc 2012 134 4163 8 10.1021/ja2095766 22295904 PMC3391577

[33] Bah A Forman-Kay JD Modulation of intrinsically disordered protein function by post-translational modifications J Biol Chem 2016 291 6696 705 10.1074/jbc.R115.695056 26851279 PMC4807257

[34] Lupas A Coiled coils: new structures and new functions Trends Biochem Sci 1996 21 375 82 10.1016/S0968-0004(96)10052-9 8918191

[35] Oishi K Okano H Sawa H RMD-1, a novel microtubule-associated protein, functions in chromosome segregation in Caenorhabditis elegans J Cell Biol 2007 179 1149 62 10.1083/jcb.200705108 18070910 PMC2140014

[36] Lu B Jin H Fu J Molecular convergent and parallel evolution among four high-elevation anuran species from the Tibetan region BMC Genomics 2020 21 839 10.1186/s12864-020-07269-4 33246413 PMC7694343

[37] Brashear WA Raudsepp T Murphy WJ Evolutionary conservation of Y Chromosome ampliconic gene families despite extensive structural variation Genome Res 2018 28 1841 51 10.1101/gr.237586.118 30381290 PMC6280758

[38] Brand SE Scharlau M Geren L Accelerated evolution of cytochrome c in higher primates, and regulation of the reaction between cytochrome c and cytochrome oxidase by phosphorylation Cells 2022 11 4014 10.3390/cells11244014 36552779 PMC9777161

