# Supplementary material for: Association analysis and in silico functional predictions of RMDN2 variants in chickens
Source: Anim Biosci. 2026 Mar 11;39(6):250758. doi: 10.5713/ab.250758 (PMC13243920; doi:10.5713/ab.250758)
Supplement: Supplementary file 1 [file ab-250758-Supplementary-1.pdf]

Supplement 1: Sequence information of primers used in this study.

**Gene id**	**Primer sequence（5′-3’）**	**Product (bp)**	**NCBI: NC_052534.1 (‘Star-Stop’)**	**XM_015283752.4 (CDS)**	**Melting temperature/(℃)**
P1	F:GGTTTGGGTAGAGTGGGA<br>R:TGTGGCAGTCTGTATTGGA	771	833-1603	Exons1	55
P2	F:CTGCATTTGGAGCACAAGGAC<br>R:AGTTCACAGAGGTAGTGCTAAACA	501	7492-7992	Exons2	60
P3	F:AAATTGATATCTGGTTCTCACGTAAA<br>R:AGATGAAAGAATGGACTGAAGTTGT	443	8728-9170	Exons3	58
P4	F:GGGGTCTGAGTGCATCTATGTAA<br>R:TGACTTCCATAAAACTACGACAAAT	405	10825-11219	Exons4	58
P5	F:TGTCGCTTAAACTTCTTGTTTCCA<br>R:CAGCACCAAACTGAACTGTGA	414	20411-20824	Exons5	59
P6	F:AGTTCTTGAGCCCTTTCA<br>R:TACATGCCCTGCTTATTT	465	22087-22551	Exons6	52
P7	F:CTCCAAAACAGAGGAGCAATCC<br>R:TTATTGTAGAGCTTTGTGGCATTT	301	29186-29486	Exons7	58
P8	F:GATCTATGGACCAGGTAA<br>R:GAGTAATAATGGGTGCTAA	363	31079-31441	Exons8 and 9	49
P9	F:AGAGCTATAAAGGGAGGG<br>R:CCAGATGTTTGAAGAGGC	388	41534-41921	Exons10	52
