# Supplementary material for: Association analysis and in silico functional predictions of RMDN2 variants in chickens
Source: Anim Biosci. 2026 Mar 11;39(6):250758. doi: 10.5713/ab.250758 (PMC13243920; doi:10.5713/ab.250758)
Supplement: Supplementary file 2 [file ab-250758-Supplementary-2.pdf]

Supplement 2: Bioinformatics software related information.

Module	Software	Website	Purpose
Functional and Structural Prediction of RMDN2	Mega 11.0	-	Constructing a phylogenetic tree
	ExPASy-ProtParam	https://web.expasy.org/protparam	Analysis of Protein and Physicochemical Properties
	ExPASy-ProtScale	https://web.expasy.org/protscale	Hydrophobicity/Hydrophilicity Analysis of Proteins
	TMHMM Serverv 2.0	http://www.cbs.dtu. dk/services/TMHMM	Transmembrane Structure Analysis of Proteins
	SignalIP5.0	https://services.healthtech.dtu.dk/services/SignalP-5.0/	Signal Peptide Prediction in Proteins
	NetnGlyc 1.0 Server	https://services.healthtech.dtu.dk/services/NetNGlyc-1.0/	Prediction of N-terminal Glycosylation Sites in Proteins
	NetPhos 3.1 Server	https://services.healthtech.dtu.dk/services/NetPhos-3.1/	Prediction of Phosphorylation Sites in Proteins
	PSorT II	https://psort.hgc. jp/form2.html	Subcellular Localization of Proteins
	SWISS-MODEL	https://swissmodel.expasy.org/interactive	Tertiary Structure of Proteins
	ProSA Web	https://prosa.services.came.sbg.ac.at/prosa.php	Evaluation of Overall Model Quality
	Uniprot[1]	https://www.uniprot.org/uniprotkb/A0A8V0XX98/entry#structure	Structural projections
	SOPMA[2]	https://npsa.lyon.inserm.fr/cgi-bin/secpred_sopma.pl	Secondary structure analysis
The Impact of Non-Synonymous Single Nucleotide Polymorphism Mutations on the Function and Structure of the RMDN2 Protein	SIFT[3]	https://sift.bii.a-star.edu.sg/www/SIFT_seq_submit2.html	Prediction of Whether Missense Mutations May Affect Protein Function
	PANTHER[4]	https://www.pantherdb.org/tools/csnpScoreForm.jsp	
	PolyPhen-2[5]	http://genetics.bwh.harvard.edu/pph2/	
	I-Mutant2[6]	https://folding.biofold.org/cgi-bin/i-mutant2.0.cgi	Evaluation of Protein Stability Post-Mutation
	mSCM[7]	https://biosig.lab.uq.edu.au/mcsm/	
	MUpro[8]	http://mupro.proteomics.ics.uci.edu/cgi-bin/predict.pl	
	PyMol-v1	-	Description of Amino Acid States Before and After Missense Mutations in the 3D Protein Structure

Note:

1. Consortium. U. UniProt: the Universal Protein Knowledgebase in 2023. Nucleic Acids Res 2023; 51:D523-31. http://doi.org/10.1093/nar/gkac1052
2. Geourjon C, Deléage G. SOPMA: significant improvements in protein secondary structure prediction by consensus prediction from multiple alignments. Comput Appl Biosci 1995; 11:681-4. http://doi.org/10.1093/bioinformatics/11.6.681

3. Ng PC, Henikoff S. Predicting the effects of amino acid substitutions on protein function. Annu Rev Genomics Hum Genet 2006; 7:61-80. http://doi.org/10.1146/annurev.genom.7.080505.115630
4. Tang H, Thomas PD. PANTHER-PSEP: predicting disease-causing genetic variants using position-specific evolutionary preservation. Bioinformatics 2016; 32:2230-2. http://doi.org/10.1093/bioinformatics/btw222
5. Adzhubei I, Jordan DM, Sunyaev SR. Predicting functional effect of human missense mutations using PolyPhen-2. Curr Protoc Hum Genet 2013; Chapter 7:Unit7.20. http://doi.org/10.1002/0471142905.hg0720s76
6. Capriotti E, Fariselli P, Casadio R. I-Mutant2.0: predicting stability changes upon mutation from the protein sequence or structure. Nucleic Acids Res 2005; 33:W306-10. http://doi.org/10.1093/nar/gki375
7. Pires DEV, Ascher DB, Blundell TL. mCSM: predicting the effects of mutations in proteins using graph-based signatures. Bioinformatics 2014; 30:335-42. http://doi.org/10.1093/bioinformatics/btt691
8. Cheng J, Randall A, Baldi P. Prediction of protein stability changes for single-site mutations using support vector machines. Proteins 2006; 62:1125-32. http://doi.org/10.1002/prot.20810
