# Supplementary material for: Association analysis and in silico functional predictions of RMDN2 variants in chickens
Source: Anim Biosci. 2026 Mar 11;39(6):250758. doi: 10.5713/ab.250758 (PMC13243920; doi:10.5713/ab.250758)
Supplement: Supplementary file 3 [file ab-250758-Supplementary-3.pdf]

Supplement 3: Polymorphisms identified in the *RMDN2* gene.

Alias	Region	Allele	Position In cDNA	Chromosomal localization	Codon	AA	Position In protein
SNP1	Exon 1	G>A	3:c.250	3:31467574	GTA/ATA	Val/ILe	84
SNP2	Exon 1	G>C	3:c.270	3:31467594	AAG/AAC	Lys/Asn	90
SNP3	Exon 2	G>T	3:c.533	3:31474246	GGG/GTG	Gly/Val	178
SNP4	Exon 2	G>A	3:c.606	3:31474319	AAG/AAA	None	202
